# The Presence of Circulating Tumor Cell Cluster Characterizes an Aggressive Hepatocellular Carcinoma Subtype

**DOI:** 10.3389/fonc.2021.734564

**Published:** 2021-10-15

**Authors:** Jing-Jing Yu, Chang Shu, Hui-Yuan Yang, Zhao Huang, Ya-Ni Li, Ran Tao, Yue-Yue Chen, Qian Chen, Xiao-Ping Chen, Wei Xiao

**Affiliations:** ^1^ Hepatic Surgery Center and Hubei Key Laboratory of Hepato-Biliary-Pancreatic Diseases, Tongji Hospital, Tongji Medical College, Huazhong University of Science and Technology, Wuhan, China; ^2^ Division of Gastroenterology, Department of Internal Medicine, Tongji Hospital, Tongji Medical College, Huazhong University of Science and Technology, Wuhan, China

**Keywords:** circulating tumor cell (CTC), circulating tumor cell clusters (CTC clusters), hepatocellular carcinoma (HCC), CellSearch™ System, prognosis, Wnt/β-catenin

## Abstract

**Background:**

Growing evidence suggests that circulating tumor cell (CTC) clusters may be an important factor in the metastatic process, but their role in hepatocellular carcinoma (HCC) remains unclear. This study aimed to characterize the molecular and clinical features of CTC cluster-positive human HCC and to assess its prognostic value in HCC patients.

**Methods:**

The CTCs and CTC clusters were evaluated in 204 HCC patients using CellSearch™ System. The counts of CTCs and CTC clusters were correlated with different clinical features, while their associations with progression-free survival (PFS) and overall survival (OS) were evaluated integrally and hierarchically by Kaplan–Meier estimates or Cox proportional regression analysis. Five cases each of CTC cluster-negative and cluster-positive patients were selected for RNA-sequencing analysis. The results of gene enrichment analysis were further verified using tissue microarray (TMA) by immunohistochemistry (IHC).

**Results:**

CTCs and CTC clusters were detected in 76 (37.3%) and 19 (9.3%) of 204 preoperative samples, respectively. CTC cluster-positive HCC represented an aggressive HCC phenotype with larger tumor size, more frequent microvascular invasion, and higher tumor stages. The survival of HCC patients utilizing CTCs and CTC clusters individually showed prognostic significance, while joint analysis revealed patients in Group III (CTC ≥ 2 and CTC cluster > 0) had the worst outcome. Stratified analysis of outcomes in Barcelona Clinic Liver Cancer (BCLC) and tumor–node–metastasis (TNM) stages indicated that patients with CTC clusters had significantly poorer prognosis in each stage than those without CTC clusters. Moreover, the RNA sequencing and TMA staining results showed that CTC cluster-positive HCCs were usually associated with Wnt/β-catenin signaling activation.

**Conclusion:**

The presence of CTC clusters characterizes an aggressive HCC subtype. CTC clusters may be used as a biomarker in predicting the prognosis on each stage of malignancy in HCC, which provides evidence for formulating therapeutic strategies for more precise treatment.

## Introduction

Tumor metastasis describes the spread of a solid tumor from the primary site to other distal organs, which diffuse through the blood circulation, and causes a worse prognosis in a variety of cancer diseases. Tumor cells that detach from the primary cancer and enter the systemic vasculature are defined as circulating tumor cells (CTCs). Many studies have verified that the number of CTCs could be used as a biomarker in the auxiliary diagnosis of cancer to evaluate the curative effects of radiotherapy and chemotherapy and to predict recurrence and metastasis in several cancers (1–4).

Clinically, CTCs have been approved by the United States Food and Drug Administration for the monitoring of metastatic breast, colon, and prostate cancer, and the National Comprehensive Cancer Network (NCCN) breast cancer guidelines (2017.v3) have included CTCs in the tumor–node–metastasis (TNM) staging system (5). In hepatocellular carcinoma (HCC), a National Institutes of Health (NIH)-sponsored clinical trial (NCT02973204) to study CTCs as a clinical auxiliary tool for HCC is underway. However, due to theoretical and technical difficulties, widespread adoption of CTCs is far from being widely used in clinical practice in HCC.

Recently, an increasing number of studies have revealed the existence of CTC clusters, consisting of multiple CTCs or CTCs surrounded by non-tumor cells, with a high metastatic potential. CTC clusters morphologically and biologically involve different phenotypes in the metastatic process. In breast cancer patients, Aceto et al. reported that many CTC clusters and high levels of tumor plakoglobin denoted adverse outcomes. In mouse models, after knockdown of plakoglobin, the formation of CTC clusters and lung metastases were suppressed (6). Murlidhar et al. showed that 50% of patients with abundant CTC clusters were detectable in early-stage lung cancer, and many larger clusters were detected in the pulmonary vein. Following long-term observation, the authors determined that the presence of CTC clusters in preoperative peripheral vein blood predicted a poor prognosis (7). CTC cluster formation was associated with disease progression and shorter survival in breast, esophageal, lung, and bladder cancer during the course of chemotherapy (8). In addition, evidence from different studies has shown that the presence of CTC clusters was associated with poorer survival in pancreatic cancer, melanoma, and colorectal cancer (9–11). Nonetheless, to date, the clinical significance of CTC clusters in HCC has been rarely reported.

In a previous study, we detected and compared the perioperative CTC counts of patients with HCC using the CellSearch™ System. The results showed that surgical liver resection was associated with an increase in CTC counts, and increased postoperative CTC numbers were associated with a worse prognosis in patients with HCC (12). In this study, we reanalyzed the data from the above study and focused on the existence and role of CTC clusters in human HCC.

## Materials and Methods

### Patients

As previously described (12), between December 2013 and June 2015, patients who had histologically confirmed HCC and had undergone liver resection were consecutively enrolled and subjected to CTC detection at the Hepatic Surgery Center, Tongji Hospital, Tongji Medical College, Huazhong University of Science and Technology. Tumor tissue samples were also collected from each patient, and the patients were continuously followed up for 3 years by counterchecking and telephoning at set intervals. In this study, 204 patients were included, and we reanalyzed their detection and clinical data. The inclusion criteria were the following (1): definitive pathological diagnosis of primary HCC (2); received curative resection, defined as complete macroscopic tumor removal (3); no prior anticancer treatment; and (4) aged between 18 and 80 years. Exclusion criteria were the following (1): with distant metastasis and (2) having other active or preexisting malignancies. The study was approved by the ethics committee of Tongji Hospital, and all patients involved provided informed consent.

### Enumeration of CTC and CTC Clusters

Preoperative peripheral blood specimens (7.5 ml) were collected 1 day before surgery. Blood collection, processing, and data analysis for CTCs and CTC clusters performed using the CellSearch™ System have been described previously (12). A CTC event is defined as an intact cell stained 4′,6-diamidino-2-phenylindole (DAPI) positive, cytokeratin positive, but CD45 negative, while a CTC cluster was defined as an aggregation of CTC containing two or more distinct nuclei and with contiguous cytoplasmic membranes (13, 14). The resulting visual images were read by two trained researchers independently.

### RNA-seq

Primary tumor tissues (stored in −80°C with RNAlater) from five cases each of CTC cluster-negative and cluster-positive HCC patients were collected for RNA-sequencing analysis. The criteria for patient selection were that there was no significant difference in the clinical parameters of patients between the two groups (**Supplementary Table S1**). A total of 1 μg RNA per sample was used as input material for the RNA sample preparations. RNA integrity was assessed using the RNA Nano 6000 Assay Kit of the Bioanalyzer 2100 system (Agilent Technologies, CA, USA). Clustering of the index-coded samples was performed on a cBot Cluster Generation System using TruSeq PE Cluster Kit v3-cBot-HS (Illumina) according to the manufacturer’s instructions. After cluster generation, the library preparations were sequenced on an Illumina Novaseq platform, and 150-bp paired-end reads were generated. Individual reads were aligned to the human genome version GRCh38.p13 using Hisat2 v2.0.5. Differential expression analysis of the two groups was performed using the DESeq2 R package (1.20.0). The resulting p-values were adjusted using the Benjamini and Hochberg’s approach for controlling the false discovery rate. Genes with an adjusted p < 0.05 identified by DESeq2 were defined as differentially expressed.

### Gene Ontology and Gene Set Enrichment Analysis

Gene Ontology (GO) enrichment analysis of all the differentially expressed genes (DEGs) was implemented using the clusterProfiler R package, in which gene length bias was corrected. GO terms with corrected p < 0.05 were considered significantly enriched by DEGs.

Gene set enrichment analysis (GSEA) of RNA-seq data was performed using GSEA v4.1.028 (http://www.broadinstitute.org/gsea/index.jsp). Gene sets were chosen from the Molecular Signatures Database (MSigDB, v7.4) and included the Hallmark gene set for pathway analysis. Gene sets upregulated in CTC cluster-positive group were ranked based on a normalized enrichment score (NES). A false discovery rate (FDR) cutoff of 25% was used to select significant gene sets.

### Tissue Microarrays and Immunohistochemistry

Cancer tissue microarrays (TMAs) contained 204 primary HCC tumor samples employed for this study. TMA sections were immune stained under the same experimental conditions. Methods for immunohistochemical (IHC) staining of tissue slides have been described previously (15). The primary antibody of β-catenin used in IHC was purchased from Proteintech, Wuhan, China (No. 51067-2-AP, 1:50 dilution).

### Statistical Analysis

The cutoff value used in prognosis was estimated using X-tile 3.6.1 software (Yale University, New Haven, CT). The results indicated that in 7.5 ml blood, a threshold CTC value of 2 or CTC cluster value of 1 showed the most significant power to predict patient outcome; then, they were used as the cutoff values in all further analyses. Chi-squared test and Fisher’s exact test were used to calculate the proportional differences among groups. Patients’ outcomes [overall survival (OS) and disease-free survival (DFS)] were estimated and compared using Kaplan–Meier survival curves and the log-rank test. The factors associated with OS and DFS were evaluated by univariate and multivariate Cox proportional hazards models. p-Values <0.05 were considered statistically significant, and all statistical analyses were performed using SPSS (version 21.0).

## Results

### The Detection of CTCs and CTC Clusters

**Figure 1:**
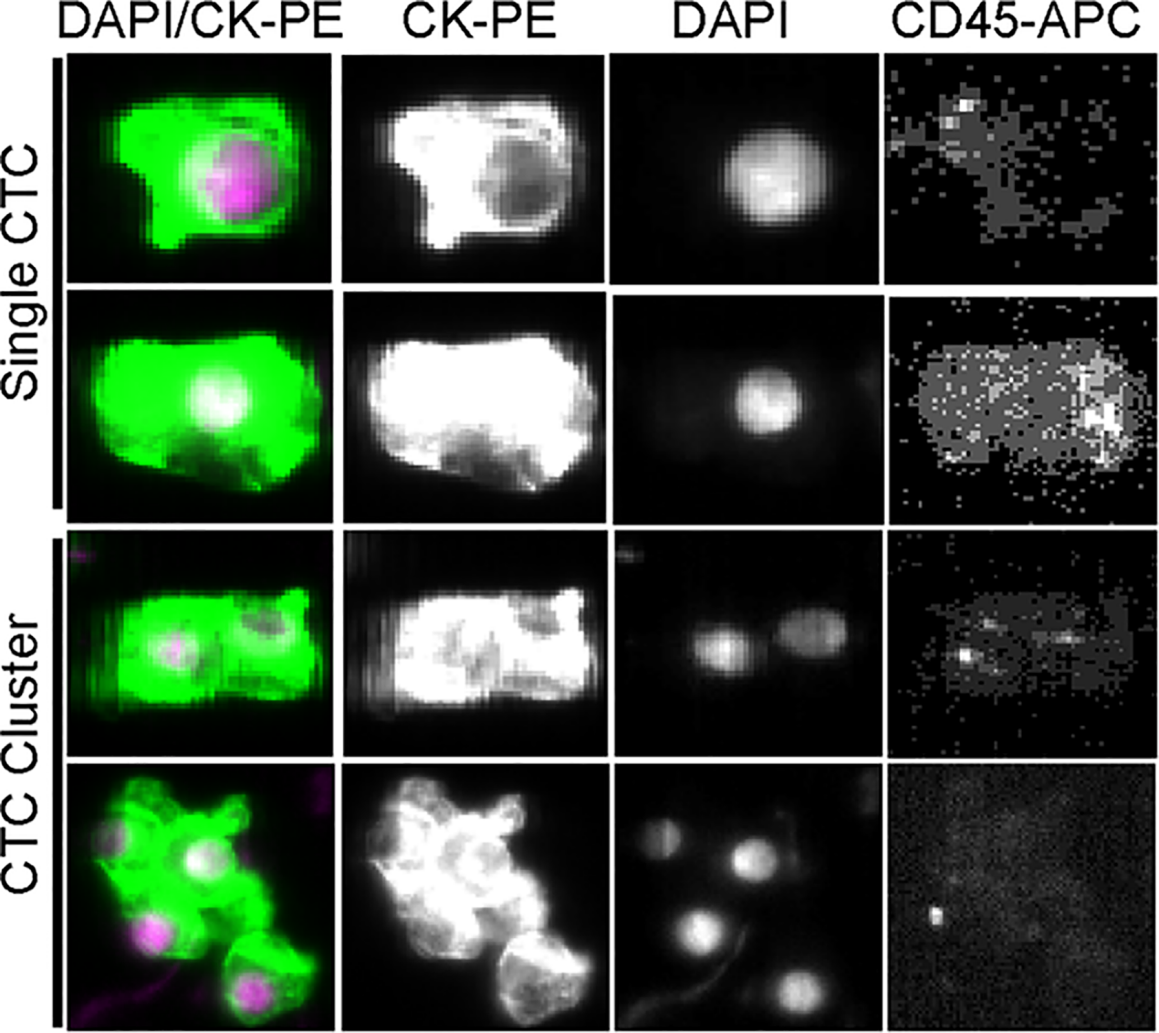
Representative images of single CTCs and CTC clusters detected in the peripheral blood of HCC patients.

The cytomorphology of CTCs and CTC clusters detected from the CellSearch™ System is shown in **Figure 1**. DAPI-positive, cytokeratin-positive, and CD45-negative cells were defined as CTCs. The aggregation of more than two CTCs formed a CTC cluster. CTCs were detected in 76 (37.3%) of 204 preoperative samples; 41 patients (20.1%) had CTC ≥2, and among them, 19 patients (9.3%) presented CTC clusters.

### Association of CTCs and CTC Clusters With Patient Characteristics

A total of 204 HCC patients were included in this study (**Table 1**). The age range of these patients was 20–80 years, and 87.3% were male. Most patients were infected with hepatitis B virus (HBV) (83.3%) and had liver cirrhosis (73.5%). The patients with CTC ≥2 showed significant differences in terms of the number of tumors, tumor size, microvascular invasion (MVI), Barcelona Clinic Liver Cancer (BCLC) stage, and TNM stage (p < 0.05). Furthermore, patients with CTC clusters were significantly associated with liver cirrhosis, tumor size, MVI, BCLC stage, and TNM stage (p < 0.05) than patients without them. The size of the largest tumor and vascular invasion were important factors affecting CTC clusters, while BCLC stage and TNM stage revealed high correlation with CTCs and CTC clusters, which indicated that CTC clusters harbored the worse degree of malignancy of HCC.

**Table 1:** Clinical characteristics of 204 HCC patients and correlation with preoperative CTCs and CTC clusters.

Clinical Characteristics	No. of Patients (N = 204)	CTCs				CTC clusters		
		CTC <2 (N = 163)	CTC ≥ 2 (N = 41)	p		CTC cluster = 0 (N = 185)	CTC cluster >0 (N = 19)	p
**Age, years**				0.463				0.169
**≤50**	109	85	24		96		13	
**>50**	95	78	17		89		6	
**Sex**				0.521				0.761
** Male**	178	141	37		161		17	
** Female**	26	22	4		24		2	
**HBsAg**				0.696				0.161
** Negative**	34	28	6		33		1	
** Positive**	170	135	35		152		18	
**Liver cirrhosis**				0.213				0.030
** No**	54	40	14		45		9	
** Yes**	150	123	27		140		10	
**Child–Pugh score**				0.166				0.298
** A**	193	156	37		176		17	
** B**	11	7	4		9		2	
**No. of tumor**				0.009				0.151
** Single**	156	131	25		144		12	
**Multiple**	48	32	16		41		7	
**Largest tumor size, cm**				0.000				0.001
**≤5**	87	83	4		86		1	
**>5**	117	80	37		99		18	
**Edmondson stage**				0.095				0.332
** I–II**	118	99	19		109		9	
** III–IV**	86	64	22		76		10	
**MVI**				0.000				0.000
** No**	151	132	19		146		5	
** Yes**	53	31	22		39		14	
**AFP, ng/ml**				0.134				0.119
** Low (<400)**	130	108	22		121		9	
** High (≥400)**	74	55	19		64		10	
**BCLC stage**				0.000				0.000
** 0–A**	74	74	0		74		0	
** B**	94	67	27		83		11	
** C**	36	22	14		28		8	
**TNM stage**				0.000				0.006
**I + II**	142	126	16		134		8	
**III + IV**	62	37	25		51		11	

### Prognosis of Patients Using CTCs and CTC Clusters

**Figure 2:**
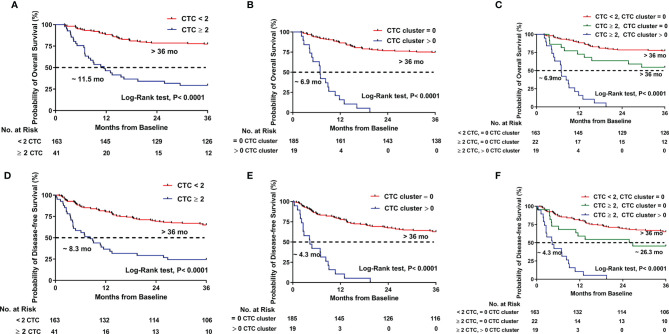
Kaplan–Meier estimates of the probability of overall survival (OS) and disease-free survival (DFS) in HCC patients. **(A)** OS between CTC ≥ 2 and CTC < 2. **(B)** OS between CTC cluster > 0 and CTC cluster = 0. **(C)** OS compared across the three risk groups (CTC < 2, CTC cluster = 0; CTC ≥ 2, CTC cluster = 0; and CTC ≥ 2, CTC cluster > 0). **(D)** DFS between CTC ≥ 2 and CTC < 2. **(E)** DFS between CTC cluster > 0 and CTC cluster = 0. **(F)** DFS compared across the three risk groups (CTC < 2, CTC cluster = 0; CTC ≥ 2, CTC cluster = 0; and CTC ≥ 2, CTC cluster > 0).

The prognostic values of CTCs and CTC clusters were evaluated individually. Patients with CTC ≥2 had significantly shorter survival than patients with CTC <2 (11.5 months *vs*. not reached, p < 0.0001 for OS; 8.3 months *vs*. not reached, p < 0.0001 for DFS) (**Figures 2A, D** **)**. Likewise, the patients existed CTC clusters also had significantly shorter survival time than those without CTC clusters (6.9 months *vs*. not reached, p < 0.0001 for OS; 4.3 months *vs*. not reached, p < 0.0001 for DFS) (**Figures 2B, E** **)**.

Then, we assessed the prognosis by using CTCs and CTC clusters jointly to see what kind of risk state might have worse outcomes. The patients were classified into three groups: Group I (n = 163) included the patients with CTC <2 but no one had a CTC cluster, Group II (n = 22) included the patients with CTC ≥2 but without a CTC cluster, and Group III (n = 19) included the patients with CTC ≥ 2 and had CTC clusters. As show in **Figures 2C, F**, patients in Group I had the longest survival time, whereas those in Group III had the shortest survival time (not reached *vs*. not reached *vs*. 6.9 months for Groups I, II, and III, respectively, p < 0.0001 for OS; not reached *vs*. 26.3 months *vs*. 4.3 months for Groups I, II, and III, respectively, p < 0.0001 for DFS).

### Multivariate Cox Proportional Regression Analysis

On univariate analysis including each preoperative variable, we found an association between outcomes (both OS and DFS) and the number of tumors, largest tumor size, BCLC stage, CTCs (≥2) and CTC clusters (>0), alpha fetoprotein (AFP) levels, MVI, and TNM stage. Next, we included these covariates into multivariate Cox proportional regression analysis and found that the existence of CTCs (≥2) and CTC clusters (>0) was an independent prognostic factor of OS (HR, 2.447; 95% CI, 1.752–3.418; p = 0.000) and DFS (HR, 1.878; 95% CI, 1.380–2.554; p = 0.000). The largest tumor size and MVI also showed highly significant associations with prognosis (**Table 2**).

**Table 2:** Univariate and multivariate Cox proportional regression analysis of factors associated with OS and DFS.

Variables	Overall survival				Disease-free Survival			
	Univariate Analysis		Multivariate Analysis		Univariate Analysis		Multivariate Analysis	
	HR (95% CI)	p	HR (95% CI)	p	HR (95% CI)	p	HR (95% CI)	p
**Preoperative variables**								
Age, >50 years *vs*. ≤50 years	0.801 (0.494–1.300)	0.370			0.742 (0.485–1.133)	0.166		
Sex, male *vs*. female	1.039 (0.515–2.098)	0.914			1.080 (0.587–1.985)	0.805		
HBsAg, positive *vs*. negative	2.013 (0.919–4.408)	0.080			2.146 (1.076–4.280)	0.030	1.848 (0.914–3.737)	0.087
Child–Pugh score, B *vs*. A	2.641 (1.204–5.794)	0.015	1.506 (0.674–3.364)	0.318	1.950 (0.899–4.228)	0.091		
No. of tumors, multiple *vs*. single	2.143 (1.292–3.554)	0.003	1.232 (0.706–2.148)	0.463	1.874 (1.194–2.940)	0.006	1.102 (0.679–1.788)	0.693
Largest tumor size, > 5 *vs*. ≤ 5 cm	6.246 (3.091–12.623)	0.000	2.738 (1.214–6.175)	0.015	4.792 (2.781–8.257)	0.000	2.773 (1.523–5.050)	0.001
BCLC stage, C *vs*. B *vs*. 0–A	3.187 (2.238–4.573)	0.000			2.475 (1.847–3.318)	0.000		
CTCs and CTC clusters, yes *vs*. no	3.563 (2.626–4.835)	0.000	2.447 (1.752–3.418)	0.000	2.831 (2.139–3.748)	0.000	1.878 (1.380–2.554)	0.000
AFP, ≥ 400 *vs*.<400	2.094 (1.295–3.385)	0.003	1.686 (0.996–2.854)	0.052	1.688 (1.106–2.577)	0.015	1.206 (0.764–1.905)	0.421
Edmonson stage, III–IV *vs*. I–II	1.220 (0.755–1.973)	0.416			1.016 (0.666–1.549)	0.942		
Liver cirrhosis, yes *vs*. no	0.955 (0. 556–1.639)	0.868			0.783 (0.493–1.245)	0.302		
MVI, yes *vs*. no	6.682 (4.088–10.922)	0.000	3.298 (1.913–5.687)	0.000	4.403 (2.862–6.773)	0.000	1.957 (1.197–3.197)	0.007
TNM stage, I–II *vs*. III–IV	3.957(2.430–6.443)	0.000			2.945(1.931–4.491)	0.000		

### CTC Clusters Predicted Stratified Outcomes in BCLC and TNM Stage

**Figure 3:**
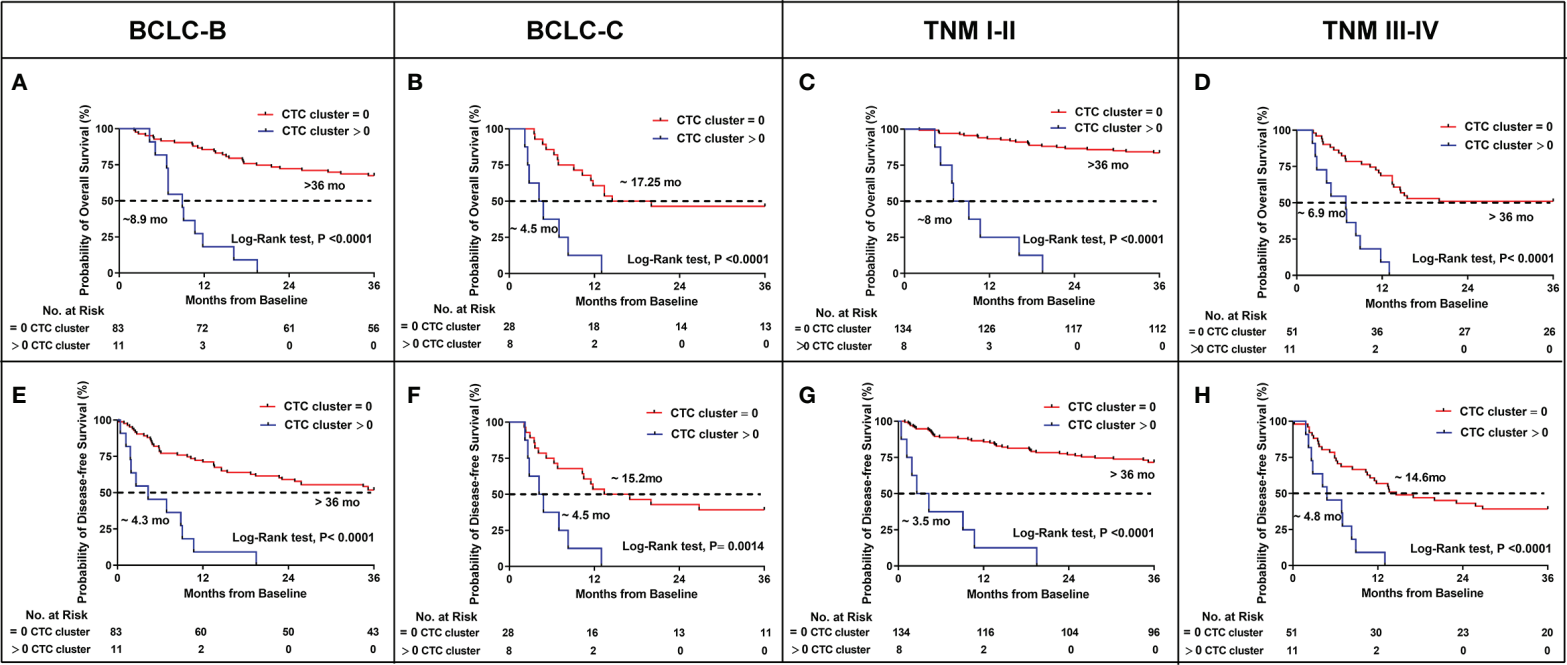
Kaplan–Meier estimates of the probability of OS and DFS stratified by CTC clusters in HCC patients between BCLC B and C, TNM I–II and III–IV. **(A)** BCLC B for OS. **(B)** BCLC C for OS. **(C)** TNM I–II for OS. **(D)** TNM III–IV for OS. **(E)** BCLC B for DFS. **(F)** BCLC C for DFS. **(G)** TNM I–II for DFS. **(H)** TNM III–IV for DFS.

Based on the above results, the presence of CTC clusters predicted the worse prognosis and was associated with BCLC and TNM stage. To confirm whether CTC clusters could predict outcomes at each stage of BCLC and TNM, we evaluated the OS and DFS in patients of stages BCLC B, BCLC C, TNM I–II, and TNM III–IV, respectively. Eleven patients with CTC clusters displayed significantly shorter OS (8.9 months *vs*. not reached, p < 0.0001) and DFS (4.3 months *vs*. not reached, p < 0.0001) among the 94 patients with BCLC B stage cancer (**Figures 3A, E** **)**. Overall, 22.22% (8/36) patients also had significantly shorter OS (4.5 *vs*. 17.25 months, p < 0.0001) and DFS (4.5 *vs*.15.2 months, p = 0.0014) compared to those without CTC clusters in BCLC stage C (**Figures 3B, F** **)**. With the presence of CTC clusters, 5.63% (8/142) of patients in TNM stage I–II and 17.74% (11/62) patients in TNM stages III–IV still had significantly shorter OS (8 months *vs*. not reached, p < 0.0001 in TNM stages I–II; 6.9 months *vs*. not reached, p < 0.0001 in TNM stages III–IV) (**Figures 3C, D** **)** and DFS (3.5 months *vs*. not reached, p < 0.0001 in TNM stage I–II; 4.8 *vs*. 14.6 months, p < 0.0001 in TNM stages III–IV) (**Figures 3G, H** **)**. We concluded that having CTC clusters indicates advanced progression similar to larger tumor size, tumor metastasis, MVI, and poorer prognosis during each stage of BCLC and TNM.

### Molecular Characteristics of CTC Cluster-Positive HCCs

**Figure 4:**
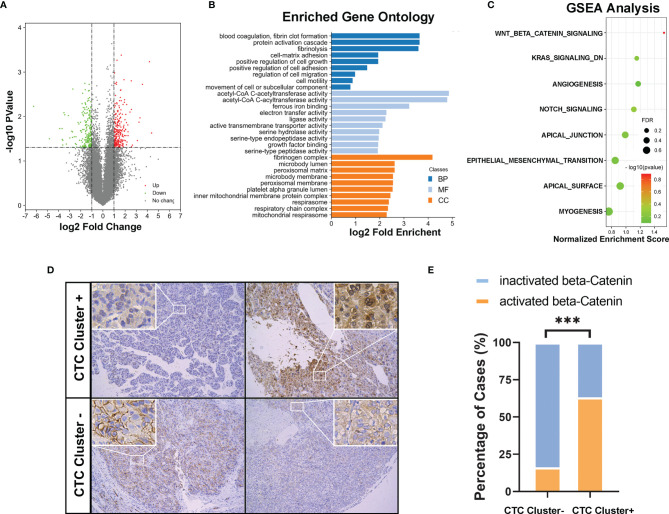
**(A)** Volcano plot showing differentially expressed genes (DEGs) in CTC cluster negative and positive HCCs (n = 5 per group). **(B)** Gene Ontology (GO) enrichment analysis of all the differentially expressed genes (MF, molecular function; BP, biological process; CC, cellular component). **(C)** GSEA results upregulated in the CTC cluster-positive group were ranked based on a normalized enrichment score (NES); FDR < 0.25 was considered statistically significant. **(D)** Representative IHC images (magnifications 100× and 200×) and **(E)** compared analysis of cytoplasmic/nuclear staining of β-catenin in CTC cluster-positive and cluster-negative clinical HCC samples. *** indicates P < 0.001.

After carefully matching clinical parameters, five cases each of CTC cluster-negative and cluster-positive HCC patients were further selected (**Supplementary Table S1**), and the primary tumor tissues were collected for RNA-sequencing analysis. In total, 182 and 131 genes were significantly up- or downregulated, respectively (fold change ≥ 2, p < 0.05, **Figure 4A**). As shown in **Figure 4B**, 28 cell signaling were significantly enriched in the GO analysis, including several cell adhesion and movement related signaling. Moreover, in the GSEA, 8/50 gene sets were upregulated in CTC cluster-positive HCCs, including Wnt/β-catenin, angiogenesis, Notch, Kras, epithelial–mesenchymal transition (EMT), and apical-related signaling. Among them, Wnt/β-catenin was considered a significant enriched gene set, with an FDR < 25% (FDR = 0.128, **Figure 4C**). To verify these findings, we next investigated the activation status of Wnt/β-catenin signaling in clinical HCC samples by IHC staining of tumor TMAs of the cohort of 204 HCCs. As showed in **Figures 4D, E**, cytoplasmic/nuclear staining of β-catenin was observed in 12 of the 19 CTC cluster-positive HCC samples (63.2%) and in only 30 of the 185 CTC cluster-negative HCC samples (16.2%). The above results suggested that, compared to CTC cluster-negative group, CTC cluster-positive HCCs showed unique molecular characteristics.

## Discussion

The prognostic evaluation of CTC clusters has been reported in several cancers in recent years, but no study has comprehensively evaluated the relevance of CTC clusters in operable HCC. In the present study, we investigated the correlation between CTCs and CTC clusters with the clinical characteristics of HCC patients and found that CTC clusters were closely related to the cancer progression. Next, we analyzed the association between CTCs, CTC clusters, and survival outcomes (OS and DFS) of HCC patients individually and jointly. Our results suggested that HCC patients with CTC clusters had a worse prognosis than those without them, whether the patients had CTCs or not. Following stratified analysis of outcomes according to BCLC and TNM stage, we confirmed that CTC clusters could be used as a biomarker in predicting the prognosis in each stage of malignancy. This may provide a basis of studying targeted strategies for precision treatment.

Wnt/Beta-catenin (β-catenin) signaling is a conserved molecular pathway that regulates hepatobiliary development and liver homeostasis (16). Abnormal Wnt/β-catenin signaling promotes the development and/or progression of different liver diseases, including cancer (17). However, the role of this pathway in liver cancer is still obscure, particularly its contributions to the tumor microenvironment and metastasis (18). With the increasing knowledge of the molecular basis of HCC, preclinical investigations and clinical trials have been conducted investigating Wnt/β-catenin signaling targeted interventions, such as with PORCN inhibitor CGX1321, FZD8 decoy receptor OMP-54F28, and LRP5/6 inhibitor Salinomycin (17). Our findings indicated that the presence of CTC clusters is closely related to the activation of this pathway in HCC. It must be acknowledged that this result has its limitations. A complex biological phenotype is usually unlikely to be caused by changes in the activity of a single gene or pathway. However, this result also suggests that the pathway does play a key role in this physiological process. Thus, further investigation is warranted to identify the selective inhibitors of Wnt/β-catenin signaling and to assess their effects in the tumor microenvironment and on metastasis.

The present study has some limitations. First, it was a retrospective reanalysis of the previous CellSearch test results. Although there have been increasing reports of CTC cluster detection using the CellSearch system (19–21), it was originally designed specifically for capturing CTC rather than CTC cluster. Second, the detection rate and amounts of CTC clusters and CTCs were not high in our study. Before the surgery, the highest counts of CTCs and CTC clusters in HCC patients’ peripheral blood were 26 and 8, respectively. The CellSearch™ System captures CTC by breaking up potential CTCs into individual cells, so that the number of CTC clusters may be greatly underestimated using this method. Compared with many other studies of patients with advanced tumors, the cases included in this study were all surgical patients, who generally had lower tumor grade and no metastases. Available evidence suggests that the distribution of CTC clusters correlates with the stage and grade of the tumor. This may be another possible reason for the lower detection rate of CTC cluster in the present study. In addition, the cells obtained from the CellSearch™ System were EpCAM positive after the preconditioning step, but increasing studies have indicated that CTC clusters and CTCs exhibited mesenchymal peculiarity (13, 14). And the EMT status of tumor cells within a CTC cluster was more evident than that of single CTC (6). The majority of isolated CTC clusters possessed the epithelial–mesenchymal phenotype in metastatic non-small cell lung cancer (NSCLC) patients, suggesting that EMT is a relevant process for metastasis caused by CTC clusters (22). Wu et al. reported that tumor metastasis was more significantly associated with the presence of mesenchymal-CTCs (M-CTCs) than with other CTC subpopulations in colorectal cancer (13). Therefore, a more efficient method to detect CTC cluster should be used in future prospective studies.

Some studies demonstrated that CTC clusters are comprised of tumor and non-tumor cells such as immune cells, platelets, leukocytes, and cancer-associated fibroblasts (23–27), which were not considered in this study. The interplay between tumor and non-tumor cells within CTC clusters was also associated with the metastasis and survival (14). CTC clusters formed by these multicellular aggregates are more likely to metastasize to distant organs by circulating freely through the veins. Au et al. demonstrated that CTC clusters could reorganize into single-file chain-like geometries promptly and reversibly, which reduced their hydrodynamic resistances. These were verified in the zebra fish model by xenotransplantation of human CTC clusters (28).

**Figure 5:**
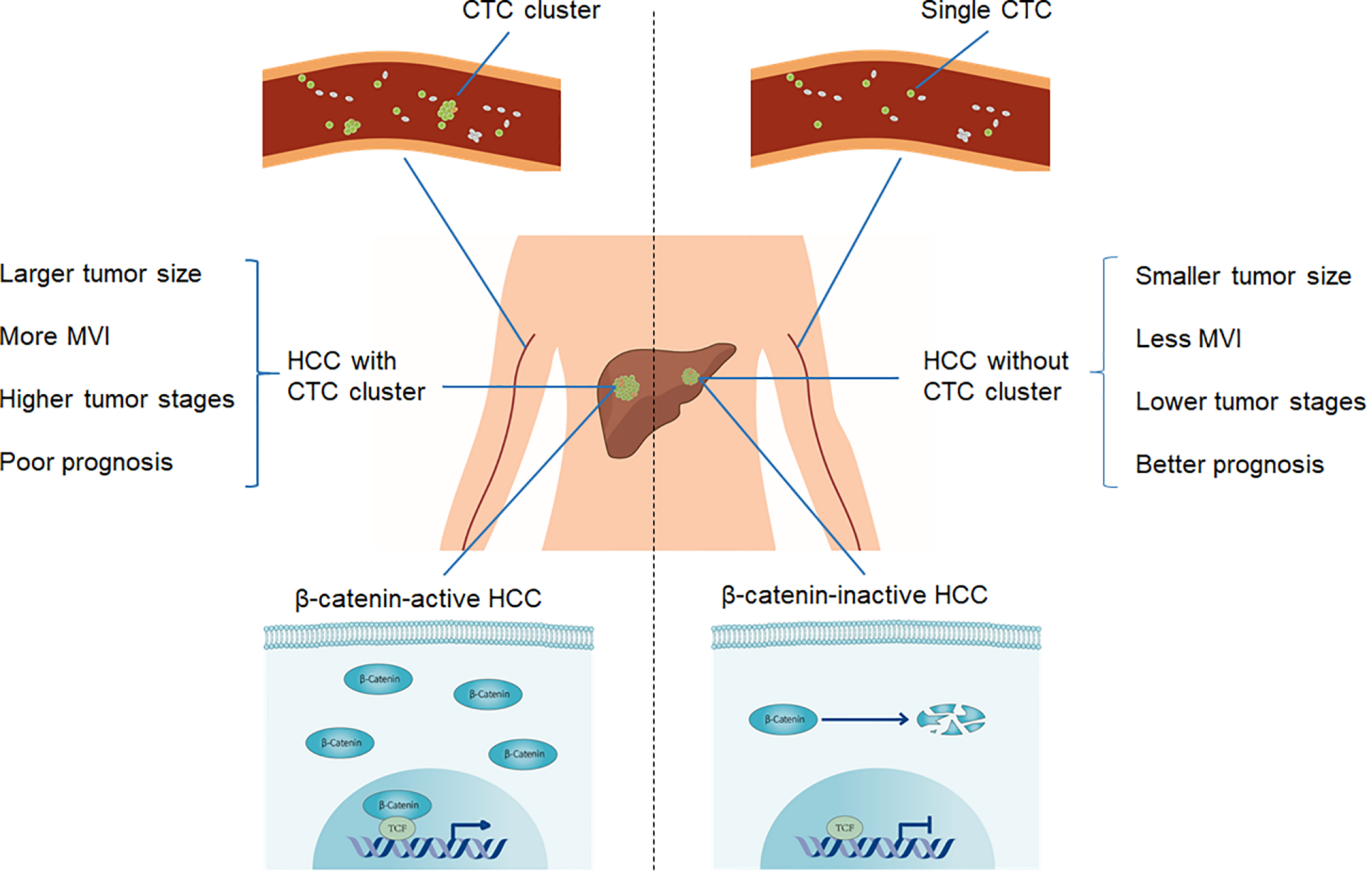
Schematic diagram of clinicopathological and molecular features of CTC clusters in HCC.

In conclusion, as summarized in **Figure 5**, we characterized CTC cluster-positive HCC as an aggressive HCC subtype, having larger tumor size, more MVI, higher tumor stages, poorer prognosis, and active Wnt/β-catenin signaling. CTC clusters may be used as a biomarker in predicting prognosis in each stage of malignancy in HCC and are worthy of further in-depth studies to define a precise management approach.

## Data Availability Statement

The RNA-seq data is available at https://www.ncbi.nlm.nih.gov/bioproject/PRJNA764061.

## Ethics Statement

The studies involving human participants were reviewed and approved by Ethical Committee of Tongji Hospital, Tongji Medical College, Huazhong University of Science and Technology (TJ-IRB20181101). The patients/participants provided their written informed consent to participate in this study.

## Author Contributions

WX, X-PC, and QC designed the project and supervised its implementation. Y-NL, RT, and Y-YC completed the sampling. J-JY and ZH read the data. H-YY performed the follow up. J-JY, CS, and WX provided the statistical analysis. J-JY and WX drafted the manuscript. All authors contributed to the article and approved the submitted version.

## Funding

This work was supported by the National Key Research and Development Program of China, No. 2016YFC0106004, and National Natural Science Foundation of China, No. 81402087.

## Conflict of Interest

The authors declare that the research was conducted in the absence of any commercial or financial relationships that could be construed as a potential conflict of interest.

## Publisher’s Note

All claims expressed in this article are solely those of the authors and do not necessarily represent those of their affiliated organizations, or those of the publisher, the editors and the reviewers. Any product that may be evaluated in this article, or claim that may be made by its manufacturer, is not guaranteed or endorsed by the publisher.
