# Supplementary material for: The Presence of Circulating Tumor Cell Cluster Characterizes an Aggressive Hepatocellular Carcinoma Subtype
Source: Front Oncol. 2021 Oct 15;11:734564. doi: 10.3389/fonc.2021.734564 (PMC8554092; doi:10.3389/fonc.2021.734564)
Supplement: Supplementary file 1 [file Table_1.docx]

**Supplementary Table 1. Clinical characteristics of 10 HCC patients selected for RNA-seq.**

| **Clinical Characteristics** | **CTC cluster=0**  **(N=5)** | **CTC cluster> 0**  **(N=5)** |
| --- | --- | --- |
| **Age, years** |  |  |
| **≤ 50** | 5 | 4 |
| **> 50** | 0 | 1 |
| **Gender** |  |  |
| **Male** | 5 | 5 |
| **Female** | 0 | 0 |
| **HBsAg** |  |  |
| **Negative** | 0 | 0 |
| **Positive** | 5 | 5 |
| **Liver cirrhosis** |  |  |
| **No** | 1 | 1 |
| **Yes** | 4 | 4 |
| **Child-Pugh score** |  |  |
| **A** | 5 | 5 |
| **B** | 0 | 0 |
| **No. of tumor** |  |  |
| **Single** | 5 | 5 |
| **Multiple** | 0 | 0 |
| **Largest tumor size, cm** |  |  |
| **≤ 5** | 0 | 0 |
| **>5** | 5 | 5 |
| **Edmondson stage** |  |  |
| **I-II** | 4 | 4 |
| **III-IV** | 1 | 1 |
| **MVI** |  |  |
| **No** | 3 | 3 |
| **Yes** | 2 | 2 |
| **AFP, ng/mL** |  |  |
| **Low (<400)** | 2 | 2 |
| **High (≥400)** | 3 | 3 |
| **BCLC stage** |  |  |
| **0-A** | 0 | 0 |
| **B** | 5 | 5 |
| **C** | 0 | 0 |
| **TNM stage** |  |  |
| **Ⅰ＋Ⅱ** | 5 | 5 |
| **Ⅲ＋Ⅳ** | 0 | 0 |
